# Body Mass Index and Nationality (Argentine vs. Spanish) Moderate the Relationship Between Internalization of the Thin Ideal and Body Dissatisfaction: A Conditional Mediation Model

**DOI:** 10.3389/fpsyg.2019.00582

**Published:** 2019-03-21

**Authors:** Silvia Moreno-Domínguez, Guillermina Rutsztein, Thomas A. Geist, Emily E. Pomichter, Antonio Cepeda-Benito

**Affiliations:** ^1^Departamento de Psicología, Universidad de Jaén, Jaén, Spain; ^2^Facultad de Psicología, Universidad de Buenos Aires, Buenos Aires, Argentina; ^3^Department of Psychological Science, The University of Vermont, Burlington, VT, United States

**Keywords:** body image, body dissatisfaction, thin ideal, eating disorders, internalization, conditional mediation

## Abstract

It is believed that Women’s exposure to Western sociocultural pressures to attain a “thin-ideal” results in the internalization of a desire to be thin that consequently leads to body dissatisfaction (BD). It is also well documented that body mass index (BMI; kg/m^2^) correlates with BD. We tested for the first time a conditional mediation model where thin-ideal *Awareness* predicted *BD* through *Internalization* of the thin ideal and the path from *Internalization* to *BD* was hypothesized to be moderated by *BMI* and *Nationality* (Argentine vs. Spanish). The model was tested with a sample of 499 young women (age = 18 to 29) from Argentina (*n* = 290) and Spain (*n* = 209). Awareness and internalization were measured with the *SATAQ-4* (Schaefer et al., 2015) and BD was measured with the *BSQ* (Cooper et al., 1987). The model was analyzed using *PROCESS* *v3.1* (Hayes, 2018). As hypothesized, thin-ideal awareness predicted BD through internalization and the path from internalization to BD was moderated by BMI and nationality. Specifically, internalization predicted BD at all level of BMI and in both samples, but the relationship between internalization and BD increased with BMI and was also stronger among Spaniards than Argentines. We argue that the findings are congruent with theories that predict that economic development and modernization contribute to normative female BD through internalization of the thin ideal and that upward social comparisons or cognitive discrepancy between self-perceived body image and the sociocultural thin ideal interacts synergistically with thin-ideal internalization to increase BD.

## Introduction

Body dissatisfaction (BD), a negative evaluation of one’s appearance and body size, is thought to be a precursor of compensatory eating disorder symptoms such as restrictive dieting, binge eating and purging, or excessive laxative use (e.g., Attie and Brooks-Gunn, 1989; Stice et al., 1998). Although BD is prevalent among individuals diagnosed with an eating disorder, in the United States and other high-income countries some degree of BD and the desire to be thinner is more often the norm than the exception among adolescent girls and women. For instance, up to 90% of women attending college in the United States may report they would like to be slimmer than they are (e.g., Williams et al., 2001); in a United Kingdom representative sample conducted by the Girlguiding organization in 2009, 93% of adolescent girls said they would like to change at least one thing about their physical appearance (as cited by Verplanken et al., 2011).

Sociocultural theorists postulate that this normative female BD is largely the result of Western (White American or Anglo-Saxon) cultural values and gendered societal norms characteristic of modern, economically developed countries (DiNicola, 1990; Brewis and McGarvey, 2000; Miller and Pumariega, 2001). In Westernized societies, physical appearance is fundamental to the value and roles given to women, the thin female body is equated with beauty and virtue, and beauty and virtue are presented as women’s path to success and life satisfaction (see Rodin et al., 1984; Stice, 1994; Stice et al., 2006). As girls and women consume mass media messages and become aware of sociocultural pressures to look thin and fit, they are likely to begin embracing and seeking (i.e., internalizing) the culturally prescribed and idealized female body (Stice, 1994). In addition to media influences, Thompson et al. (1999) postulated that peer and family pressure also contribute to women’s internalization of the thin ideal. This internalization of the thin ideal is believed to cause BD as women compare themselves unfavorably against their unrealistic body-image goals (e.g., Moreno-Domínguez et al., 2018).

There is considerable research linking the consumption of media exposure to BD (e.g., Bessenoff, 2006; Corning et al., 2006; Grabe et al., 2008; Bonafini and Pozzilli, 2011), and experimentally controlled exposure to mainstream media images of thin models has been repeatedly shown to increase BD in young women (e.g., Bessenoff, 2006; Moreno-Domínguez et al., 2018; see also Want, 2009). Moreover, the hypothesis that the internalization of the thin ideal mediates the relationship between awareness of the cultural ideal and body dissatisfaction has considerable empirical support (e.g., Thompson et al., 1999; Stice, 2002; Fingeret and Gleaves, 2004; Warren et al., 2005; Shin et al., 2017; for a review see López-Guimerà et al., 2010). In addition, empirical research suggests that sociocultural pressure to adhere to the thin ideal may lead to substantive BD only in individuals who report high levels of thin ideal internalization (Dittmar and Howard, 2004; Dittmar et al., 2009; López-Guimerà et al., 2010). Correlational research further suggests that internalization of the thin ideal, rather than mere awareness of the ideal, is critical for the development of BD. That is, correlations between internalization of the thin ideal and BD are consistently and often twice the size of reported correlations between mere awareness of the thin ideal (or perceived external pressure to be thin) and BD (see Cafri et al., 2005).

The theory that Western values create a sociocultural environment that fosters female BD would predict that a population’s level of BD should correlate with the degree to which that population’s sociocultural milieu is, or becomes, Westernized. This prediction is congruent with the observation that along with the growing global influence of American sociocultural values in the world (Mann, 2001), the eating disorders literature has progressively reported an increasing prevalence of female BD and disordered eating in non-Western countries (e.g., Forbes et al., 2012; Schulte and Thomas, 2013; Choi and Choi, 2016). Nonetheless, we also note that the effects of Western culture exposure and Western acculturation on eating-disorder risks could be heterogeneous and may differ considerably across ethnically, culturally or nationally diverse groups (Becker et al., 2010) and across diverse samples from the same population (e.g., Warren et al., 2005). Additionally, culturally unique protective factors can also differentially impact the internalization-mediated effect of awareness of sociocultural pressure to be thin on BD (Warren et al., 2005). Thus, it would be important to test across culturally diverse samples the theory that women’s exposure to Western sociocultural pressures to be thin results in the internalization of a desire to be thin that consequently leads to BD.

Forbes et al. (2012) compared social pressure to be thin, internalization of the thin ideal, body-dissatisfaction and eating disorder symptoms across undergraduate women from Argentina, Brazil and the United States Their results revealed that the United States sample reported significantly greater awareness and internalization of the thin ideal, higher discrepancy between their perceived vs. their desired body image (a measure of BD), and more eating disorder symptoms than the Argentine and Brazilian samples. Although Forbes et al. (2012) speculated that “cultural aspects” may “protect” Argentine and Brazilian women from internalizing the “thin ideal,” these authors could not explain how culture prevented the internalization of the thin ideal.

Research conducted with Argentine and Spanish samples suggest that women from both countries present with considerable levels of normative BD, which could be driven, at least in part, by the internalization of social and cultural pressure to adhere to the Western, thin female ideal. For example, up to 80% of adolescent girls from a Buenos Aires sample reported being dissatisfied with their weight (McArthur et al., 2005). Similarly, many studies have reported strong evidence of normative BD (e.g., Gleaves et al., 2000; Warren et al., 2005) and internalization of the female thin ideal among Spaniards (Lameiras Fernández et al., 2003; De Gracia et al., 2007; Ramos Valverde et al., 2010; Ramos et al., 2016). We only know of one study that has compared BD across Argentines and Spaniards (Marrodán et al., 2008). These authors found that whereas both Argentine and Spanish adolescent girls reported a general desire to be thinner, Spanish participants reported lower self-image satisfaction than the Argentines. In a study that compared Argentine and Swedish adolescent girls, Swedes were more likely than Argentines to report being “too fat” (Holmqvist et al., 2007). Independent of BMI, these authors found that among those who self-classified as being too fat, Swedish girls reported higher body weight and body appearance dissatisfaction than Argentine girls (Holmqvist et al., 2007).

Given the paucity of research on BD comparing Argentinian and Spanish samples, we can only theorize about the extent to which Western values of female thinness may have differentially impacted women in two Spanish-speaking countries with different sociocultural and economic milieus. For example, if within a given population normative BD increases with the population’s degree of exposure to modernization and economic development (DiNicola, 1990), we can hypothesize that normative BD and the underlying processes that lead to its manifestation should be more entrenched among Spaniards than Argentines. That is, we assume that historically Spain has had a longer and more established exposure to modernization and high economic development. For instance, the World Bank has classified Spain as a “high-income” country every year since they began to classify countries in 1988 by Gross National Income per capita, while Argentina was assigned that rank only in 2015 and 2018 (World Bank Data Help Desk, 2018). Therefore, we hypothesize that “nationality” (Spanish vs. Argentine) may function as a moderator of the association between the internalization of the thin ideal and its effect on BD, as it seems to have been the case in the comparison between Swedish and Argentine girls who self-classified as being “too fat” but differed on the extent to which they were dissatisfied with their body appearance and body weight (Holmqvist et al., 2007).

Additionally, although exposure to the thin ideal may cause BD through an internalization process, BD is a complex construct that may develop differently not only from culture to culture, but also across individuals. That is, when we assert that internalization of the thin ideal leads to BD we should not ignore the possibility that women may differ on the extent to which this process is manifested. For instance, BMI correlates with (McCabe and Ricciardelli, 2003; Petrie et al., 2010; Laus et al., 2011) and prospectively predicts (e.g., Stice and Whitenton, 2002; Bearman et al., 2006; Clark and Tiggemann, 2008; Wojtowicz and von Ranson, 2012; Quick et al., 2013) BD in adolescent girls. BMI is also consistently associated with BD in samples of college students and adult women (e.g., Bulik et al., 2001; Williams et al., 2001; Warren et al., 2005; Dalley et al., 2009), and there is empirical support suggesting BMI and BD share risk factors, including heritability (Wade et al., 2011). Given the well documented association and predictive relationship between BMI and BD, and the often tested and supported hypothesis that BD is largely caused by a process of social comparison whereby women repeatedly evaluate themselves negatively against the socially prescribed thin ideal (see Want, 2009), we posit that BMI likely moderates the relationship between the internalization of the thin ideal and BD. That is, if cognitive self-discrepancy between one’s ideal and perceived body image is the underlying mechanism that links internalization to BD (e.g., Bessenoff, 2006), it is reasonable to hypothesize that among individuals with similar levels of internalization, those with higher BMIs are more likely to experience greater self-discrepancy and thus higher BD than individuals with lower BMIs.

**FIGURE 1:**
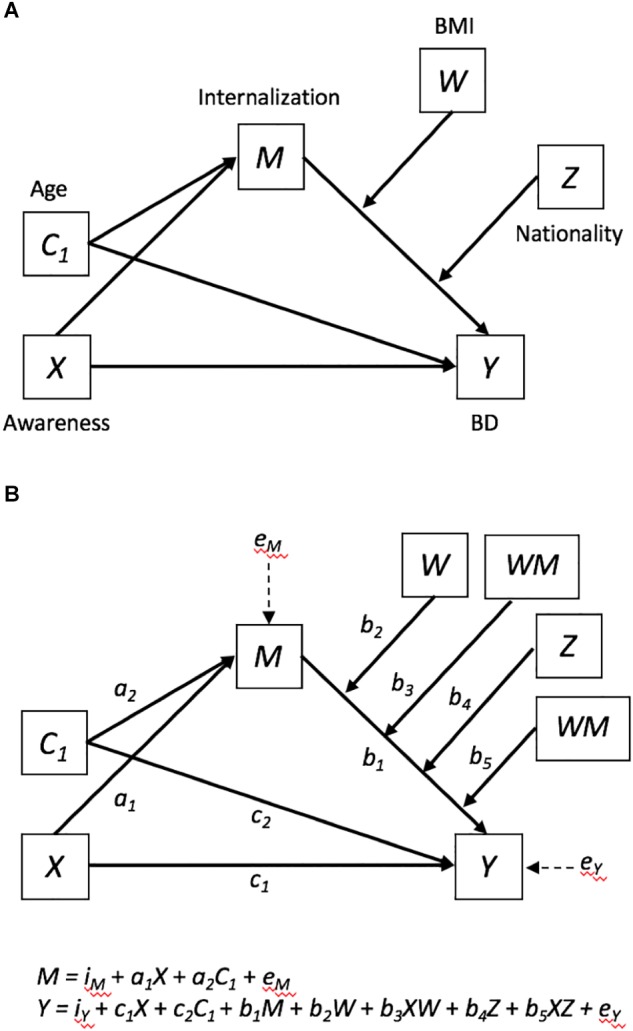
**(A)** Conceptual *Conditional* *Mediation* *Model* such as that *Awareness* of the thin ideal (*X*) predicts *Body* *Dissatisfaction* (*BD*) directly (*X* → *Y*) and indirectly through Internalization of the thin ideal (*X* → *M*; *M* → *Y*). The model is conditional on Body Mass Index (BMI; *W*) and nationality (*Z*). *Age* (*C_1_*) is a covariate. **(B)** Depicts the statistical model described in panel **(A)**.

Our hypothesized model is depicted in Figure 1, the conceptual model is depicted in panel A and the statistical model, with Age as a covariate, is depicted in panel B. We used an Argentine and a Spanish sample of young women to test a conditional mediational model that predicts that the effects of awareness of the thin ideal (*X*) on BD (*Y*) are mediated through internalization of the thin ideal (*X* →*M* → Y). We further specify that the indirect effect component of internalization to BD (*M* →*Y*) is conditional on BMI (*W*) and Nationality (*Z*). We directionally predicted that whereas the mediational model would replicate across countries, the paths *MW* →*Y* and *MZ* →*Y* would be significant. Examination of the interaction effects should reveal that the *M* →*Y* effect is more pronounced among participants with higher BMIs, as well as among Spaniards than Argentines.

## Materials and Methods

### Participants

We used a convenience sample of 499 young women (age = 18 to 29) recruited from the student populations from the University of Buenos Aires, Argentina (*n* = 290) and the University of Jaen, Spain (*n* = 209). Research assistants recruited participants by making in person announcements at the beginning or end class periods requesting female volunteers to participate in a survey study about body image. Uncompensated volunteers attending group sessions of 30 to 40 students held in a large classroom hall read and signed the informed consent form and completed the questionnaires. Research assistants asked the participants to work individually and respond to the items without overthinking their answers, letting them know that there were neither correct nor incorrect answers as different women may have different experiences, perceptions or feelings about questions asked. The internal review boards of both universities approved the study as being compliant with the American Psychological Association’s ethical principles for conducting research with human participants.

Means, standard deviations and *t-*test comparisons between the two samples for Age, BMI, *Body* *Satisfaction* *Questionnaire* (BSQ, Cooper et al., 1987) and the Awareness and Internalization scales of the *Sociocultural* *Attitudes* *Toward* *Appearance* *Questionnaire-4* (SATAQ-4; Schaefer et al., 2015) scores are presented in Table 1.

**Table 1:** Means, standard deviations, and two-tailed *t-*test comparisons between the Argentine and Spanish samples for age, body mass index (BMI), thin ideal awareness (SATAQ-4A), thin ideal internalization (SATAQ-4I), and body dissatisfaction (BSQ).

	Spaniards (*n* = 209)		Argentines (*n* = 290)			
	*M*	*SD*	*M*	*SD*	*t* (497)	*p* *<*
Age	22.1	2.0	24.7	2.0	14.51	0.0001
BMI	22.59	3.83	22.35	3.88	-0.60	0.5526
SATAQ-4A	25.97	9.93	25.53	9.89	-0.48	0.6338
SATAQ-AI	25.45	9.50	24.43	9.51	-1.15	0.2509
BSQ	87.02	42.11	74.13	29.90	-3.95	0.0001


With regards to Age and BMI, the Argentinian sample was about 2 ½ years and significantly older (*M* = 24.7; *SD* = 2.0) than the Spanish sample (*M* = 22.1; *SD* = 2.0), but the average BMI (kg/m^2^) was similar across Argentines (*M* = 22.35; *SD* = 3.88) and Spaniards (*M* = 22.59; *SD* = 3.83). Average BSQ scores were well below clinical levels in both samples but Spaniards scored significantly higher (*M* = 87.02; *SD* = 42.11) than Argentines (*M* = 74.13; *SD* = 29.90). Spaniards and Argentines scored similarly on the Awareness (*M* = 25.97; *SD* = 9.93 vs. *M* = 25.53; *SD* = 9.89) and Internalization (*M* = 25.45; *SD* = 9.50 vs. *M* = 24.43; *SD* = 9.51) scales of SATAQ-4.

### Measures

#### BMI

Participants reported their weight in kilograms and height in meters. The BMI was calculated using the formula kg/m^2^. Whereas actual measurements of height and weight should yield more accurate estimates of BMI, in our experience BMIs calculated from either self-reported height and weight, or from measured height and weight are highly correlated (*r_2_* and/or *r* *>* 0.98) and do not change found relationships between BMI and other variables (see also Olfert et al., 2018).

#### BSQ

The BSQ asks participants to report how they feel about their appearance by answering 34 items that are scored in a six-point, Likert-type scale from *never* (1) to *always* (6). Total scores can range from 34 to 204, with scores above 104 being in the clinical range. The BSQ assesses general preoccupation with body shape and size, and fears of becoming or feeling fat. The measure has been shown to yield excellent reliability indices, to discriminate between eating disorder samples and controls, and to be sensitive to treatment gains (Cooper et al., 1987; Zabinski et al., 2001). Several investigations have used the Spanish version of the BSQ and reported adequate reliability and validity (e.g., Warren et al., 2008; Moreno et al., 2009). In the present study, internal reliability for the Argentinian (α = 0.96) and Spanish (α = 0.98) scores were excellent.

#### SATAQ-4

The SATAQ-4 asks respondents to rate their agreement with 22 statements using a 5-point Likert-type scale from 1 (“definitely disagree”), to 5 (“definitely agree”). The SATAQ-4 can be divided into two scales, one corresponding to 12 items that ask about being aware of perceived pressure from family, peers, and the media to look thin or athletic (awareness/pressure scale; heretofore SATAQ-4A) and one corresponding to 10 items that ask about acceptance or self-imposed pressure to look thin or athletic (internalizing scale; heretofore SATAQ-4I). Psychometric evaluations of the SATAQ-4 report evidence of convergent validity with various eating disorder and body dissatisfaction measures, as well as high internal consistency scores in samples of English (Schaefer et al., 2015) and Spanish (Llorente et al., 2015) speaking college students. In our sample, internal consistency was excellent for both the SATAQ-4A and SATAQ-4I scores in both samples (α = 0.89 to 0.91).

#### Analytic Approach

*Mediation* analysis allows investigators to test *how* an independent variable *X* exerts a significant indirect effect on a dependent variable *Y* by changing a third variable *M*, which in turn causes changes on *Y* (*X* →*M* → Y; see Figure 1A). One of the most common methods used to test mediation models is the *causal-steps* strategy (Baron and Kenny, 1986), which despite of its popularity has important conceptual and power limitations (see MacKinnon et al., 2002; Preacher et al., 2007). Conceptually and mathematically sound alternatives to the causal-steps method include *resampling* *or* *bootstrapping* and *product* *of* *coefficients* strategies (Preacher and Selig, 2012; Hayes, 2018). In its simplest form, *moderation* analysis examines *when* the relationship between a predictor X and a predicted outcome Y depend, or is conditional on, a third variable, or moderator W*;* a question that is probed by testing whether the regression weight of Y on X varies systematically as a function of W (i.e., whether the hypothesized moderator W interacts with X to predict Y). *Moderated* *mediation* analysis simultaneously tests ***how*** and ***when*** a relationship between an antecedent X and an outcome Y occurs (see Hayes, 2018). Specifically, moderated mediation occurs when one or more relationship paths between X, M, and *Y* (i.e., *X* →*M*, *X* →*Y*, or *M* →*Y*) in a mediation model (*X* →*M* →*Y*) interact with one or more moderators (e.g., *XW* →*M*, *MW* →*Y*, *MZ* →*Y*, etc.) such that the relationship between the antecedent and the consequent (e.g., *M* → *Y*) is contingent on the level of the moderator (see Preacher et al., 2007; Hayes, 2018).

Preacher, Hayes and their colleagues have provided various “macros” for SPSS and SAS that ease the task of carrying out mediation and moderation analyses (e.g., Preacher and Hayes, 2004, 2008; Preacher et al., 2007; Hayes and Matthes, 2009). These and other similar tools have been integrated in *PROCESS*, a comprehensive tool that simplifies the testing of mediation, moderated and moderated (conditional) mediation models (Hayes, 2013, 2018). PROCESS uses path analysis modeling with ordinary least squares (OLS) and logistic regression. We used SPSS v24.0 and the most recently available version of the macro, PROCESS v3.1 (Hayes, 2018). To specify the statistical model, we mean-centered the variables forming the interaction terms (i.e., M, W, and Z), selected heteroscedasticity consistent standard errors (HC1; see Hayes and Cai, 2007), and specified 10,000 iterations to estimate bootstrap 95% coefficient confidence intervals (*CI*s; see Hayes, 2018). The use bootstrapping to calculate *CI*s does not assume normally distributed indirect effects, provides a more accurate test of mediation effects than the causal-steps Sobel test (see Preacher et al., 2007), and allows for pairwise comparisons between coefficients of an antecedent variable at different values of a moderator (see Hayes, 2018). PROCESS also facilitates the visual inspection of significant interactions with hypothesized moderators by producing and plotting the predicted values of an outcome variable regressed on the predictor at different values of the moderator (Hayes, 2018). For interpretation, PROCESS provides standard errors, *p*-values, confidence intervals for the direct effect coefficients, and bootstrap confidence intervals for conditional indirect effects and for conditional indirect effects pairwise contrasts. *CI*s that do not straddle zero are indicative of statistical significance.

For our analyses we used PROCESS v3.1, Model 16, which allowed us to test a moderated mediation model such that BD (*Y*) was regressed on Awareness of pressure to attain the Western ideal of beauty (*X*) and the covariate Age (*C*_1_) through the Internalization of the ideal (*M*) (see Figure 1). Model 16 further specifies that the effect from the hypothesized mediating or antecedent variable *M* (Internalization) to the outcome or consequent variable *Y* (BD) is conditional on two moderating variables *W* (BMI) and *Z* (Argentines [0] vs. Spaniards [1]). Significant interactions (Internalization by BMI; Internalization by Nationality) were examined by visualizing predicted values of BSQ scores at the 16th, 50th and 84th percentiles of SATAQ-4I centered and BMI centered scores within the Argentine, as well as the Spanish sample (see Hayes, 2018).

## Results

Table 2 displays the model’s summary information (see Figure 1), including the regression coefficients, standard errors, and *p*-values. The results were congruent with the hypothesis that greater awareness of the thin ideal increases internalization of the ideal (*a*_1_ = 0.3568; *p* < 0.0001). This finding was evident while controlling for the effects of age, which was in turn negatively associated with self-reported internalization of the thin ideal (*a_2_* = -0.5632; *p* < 0.0013). That is, younger age was a risk factor for internalization of the thin deal. The results also supported the hypothesized and often reported finding that internalization contributes to BD, as the coefficient for internalization was significantly greater than zero (*b_1_* = 2.0597, *p* < 0.0001), even while controlling for the effects of awareness, age, BMI, nationality, and the interaction terms formed by the product of internalization by BMI, and internalization by nationality (see column “Y (BSQ)” in Table 2). It should be noted that awareness (*c_1_* = 0.8771; *p* < 0.0001) and BMI (*b_2_* = 2.4598; *p* < 0.0001) were also significant predictors of BD, but neither age (*c_2_* = -0.2009; *p* < 0.0001) nor nationality (*b_4_* = 3.9007; *p* < 0.1492) predicted BD. Importantly, the interactions BMI by internalization (*b_3_* = 0.908; *p* < 0.0094) and nationality by internalization (*b_5_* = 0.6423; *p* < 0.0143) were statistically significant, meaning both BMI and nationality moderated the relationship between internalization and BD.

**Table 2:** Regression results for the conditional mediation model depicted in Figure 1 while controlling for age.

		Consequent						
		*M* (SATAQ-4I)				*Y* (BSQ)		
Antecedents		Coefficient	SE (HC1)	*p<*		Coefficient	SE (HC1)	*p<*
*X* (SATAQ-4A)	*a_1_*	0.3568	0.0414	0.0001	*c_1_*	0.8771	0.1642	0.0001
*M* (SATAQ-4I)		——	——	——	*b_1_*	2.0597	0.1459	0.0001
*W* (BMI)		——	——	——	*b_2_*	2.4598	0.6197	0.0001
*M* *×W*		——	——	——	*b_3_*	0.0908	0.0347	0.0094
*Z* (Arg = 0; Spa = 1)		——	——	——	*b_4_*	3.9007	2.6989	0.1492
*M* *×Z*		——	——	——	*b_5_*	0.6423	0.2610	0.0143
*C*_1_ (Age)	*a_2_*	-0.5632	0.1727	0.0013	*c_2_*	-0.2009	0.5730	0.7262
*Constant*	*i_M_*	3.9121	4.2854	0.3620	*i_Y_*	61.5878	14.2620	0.0001
	*R*^2^ = 0.425				*R*^2^ = 0.783			
	*F*(2, 360) = 48.7564; *p* < 0.0001				*F*(7, 355) = 82.7053; *p* < 0.0001			


**FIGURE 2:**
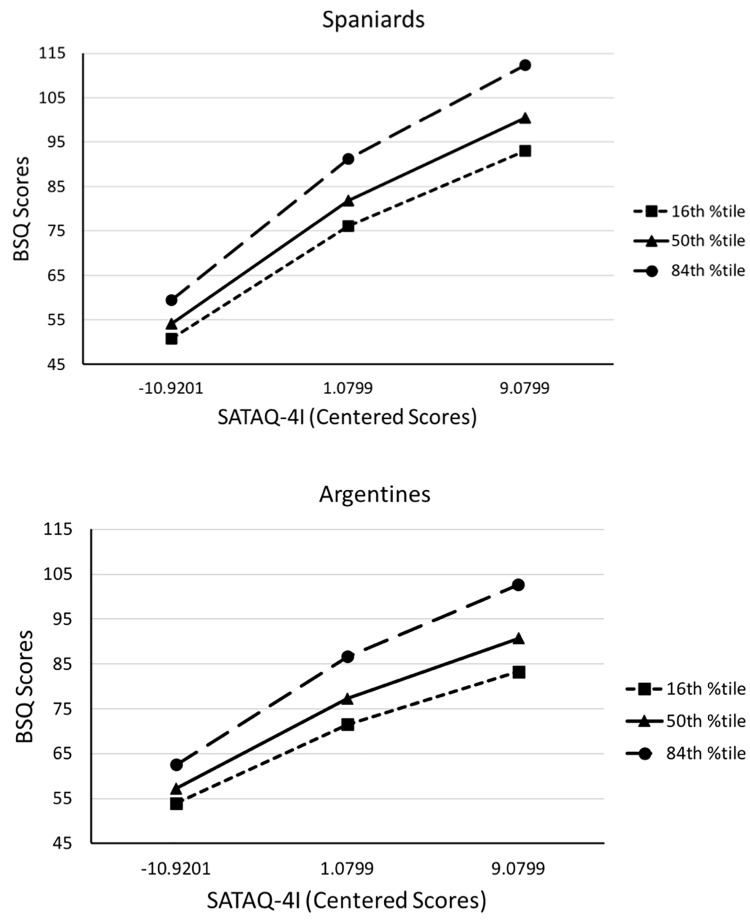
Visual representation of the association between internalization (SATAQ-AI centered scores) and Body Dissatisfaction (BSQ scores) at each of the probed BMI values in the Spanish and Argentine samples.

**FIGURE 3:**
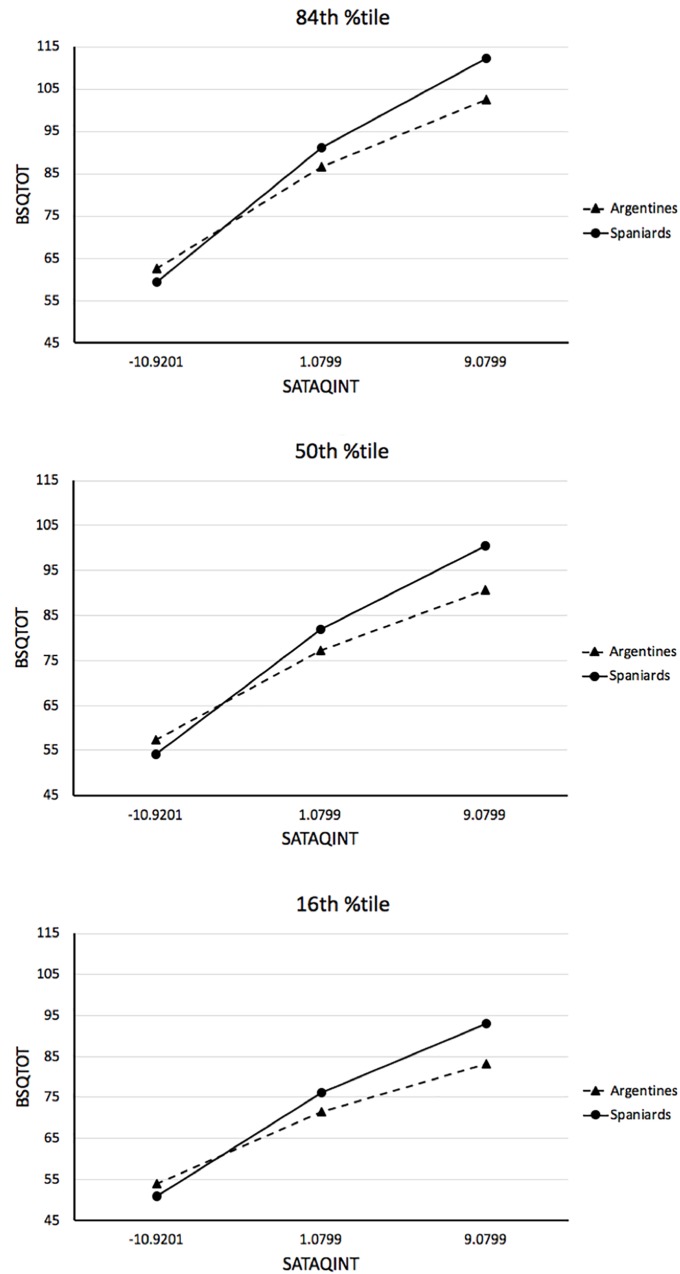
Visual representation of the association between internalization (SATAQ-AI centered scores) and Body Dissatisfaction (BSQ scores) in the Spanish *vis-à-vis* the Argentine sample at each of the probed BMI values.

Significant interactions in the OLS regression model require further scrutiny. To this effect, PROCESS generates an *Index* *of* *Moderated* *Mediation* with bootstrap *CI*s that indicate how much the mediated effect of awareness on BD through internalization change by each unit of increment in the corresponding moderator. We found that both BMI and nationality significantly moderated the indirect effect of awareness on BD through internalization of the thin ideal. That is, neither the bootstrap 95% *CIs* for the index of moderation for BMI, (*Index* = 0.0324; *CI* [0.0076, 0.0618]) nor for nationality (*Index* = 0.2292; *CI* [0.0442, 0.4335]) straddle zero (see Table 3). These results mean that the association between Internalization and BD was significantly stronger at larger BMI values (see Figure 2) and more pronounced among Spaniards than Argentines (see Figure 3).

**Table 3:** Indices of moderated mediation for BMI and nationality and conditional indirect effects for the Argentine and Spanish samples at the 16, 50, and 84th percentiles of the distribution of BMI scores.

Indices of moderated mediation					
		**Index**	**SE**	**LLCI**	**ULCI**

Body max index (kg/m^2^)		0.0324	0.0138	0.0076	0.0618
Nationality (Arg = 0; Spa = 1)		0.2292	0.0986	0.0442	0.4335

**Conditional indirect effects**					
**BMI Percentile**	**Nationality**	**Effect**	**SE**	**LLCI**	**ULCI**

16th	Argentine	0.5230	0.0937	0.3492	0.7153
16th	Spanish	0.7522	0.1266	0.5212	1.0162
50th	Argentine	0.5963	0.0965	0.4170	0.7954
50th	Spanish	0.8254	0.1240	0.5973	1.0847
84th	Argentine	0.7137	0.1193	0.4963	0.9665
84th	Spanish	0.9428	0.1357	0.6942	1.2289


PROCESS also allows to probe significant conditional (moderated) indirect effects at each of different moderator values. In our case, we probed the relationship between internalization and BD for the Argentines (0) and Spaniard (1) participants at BMIs corresponding to the 16, 50, and 84th percentiles. Figure 2 provides the visual representation of the conditional indirect effects at each of the probed BMI values in both samples. Figure 3 gives a visual representation of the conditional indirect effects of Argentines *vis-à-vis* the Spaniards for each of the BMI values. Table 3 shows that all of the conditional indirect effects with bootstrap *CI*s for the Argentine and Spanish samples at each of the three probed BMI values were significant as none of the *CI*s straddle zero. Table 4 further shows pairwise contrasts that tested for conditional indirect effect differences across probed BMI values. Each of the contrasts tested was statistically significant as none of the *CI*s straddle zero.

**Table 4:** Pairwise conditional indirect effect contrasts, standard errors, and 95% *CIs* across BMI percentile values within nationalities.

BMI %tiles	(Effect 1-Effect 2)
(nationality)	= Contrast	SE	LLCI	ULCI
50th vs. 16th (Argentine)	(0.5963-0.5230) = 0.0732	0.0313	0.0173	0.1396
50th vs. 16th (Spanish)	(0.8254-0.7522) = 0.0732	0.0313	0.0173	0.1396
84th vs. 50th (Argentine)	(0.7137-0.5963) = 0.1174	0.0501	0.0277	0.2239
84th vs. 50th (Spanish)	(0.9428-0.8254) = 0.1907	0.0814	0.0449	0.3635


## Discussion

We found robust support for the hypothesis that awareness of pressure from media, family and friends to look thin and athletic predicts BD dissatisfaction, and that this effect is partially channeled through the effect of internalization of the ideal on body dissatisfaction. That is, awareness of the thin ideal or SATAQ-4A scores predicted internalization of the thin ideal (SATAQ-4I scores), and both awareness and internalization scores predicted BD (BSQ scores) while controlling for each other and the model’s covariates and moderators: age, BMI, nationality, BMI by internalization and nationality by internalization. The results are thus congruent with the belief that sociocultural milieus that promote an idealized standard of female beauty and virtue characterized by an unrealistically thin or athletic body shape foster female BD (e.g., Stice et al., 2006). This pressure to conform to the thin ideal of beauty originates from various sources, including family members, and peer groups (Thompson and Stice, 2001), although mass media channels are thought to be particularly powerful in promoting these ideals (Tiggemann et al., 2000; Shin et al., 2017).

We anticipated that the effects of awareness of the thin ideal on BD would be mediated by internalization of the ideal in both Argentines and Spaniards, and we also predicted that nationality would interact with internalization such that the association between internalization and BD would be more pronounced in Spaniards than Argentines. We based our prediction on the assumption that the Spanish sample belonged to a more economically modernized population than the Argentine sample (see DiNicola, 1990), an assumption we based on longstanding, *per* *capita* income differences between the two countries (World Bank Data Help Desk, 2018). The significant regression coefficient of the interaction between internalization and nationality, as well as the bootstrap *CI* of the index of moderated mediation support our directional prediction. Our results clearly show that the relationship or regression slope between internalization and BD was more pronounced in the Spanish than Argentine sample across participants with low, average and high BMIs. We note here also that the Spanish and Argentine sample did not significantly differ on awareness of the thin ideal, internalization of the thin ideal, or BMI. However, the Spanish sample reported significantly higher levels of BD that the Argentine sample, and Spaniards were significantly younger than the Argentines. Given that BD was regressed on awareness and internalization of the ideal while controlling for age, BMI and nationality, the interaction between nationality and internalization explains why BD scores were higher among Spaniards than Argentines. Thus, whereas it is not unique that internalization of the thin ideal mediated the predicted positive relationship between awareness of the thin ideal and BD (e.g., Warren et al., 2005), the replication of the model in a Latin American sample, and the demonstration that the model explains differences in average BD between two Spanish speaking samples from different continents with marked economic development differences represents a theoretically important and novel contribution to the literature.

We reasoned that the effect of internalization of the thin ideal on BD could differ not only across cultures (e.g., Warren et al., 2005) but also as a function of individual differences. Specifically, we predicted that BMI would moderate the relationship between internalization and BD. Our results support this prediction as evidenced by the significant interaction between internalization and BMI, the CI of the index of moderation of the indirect effect, and the significant pairwise contrast between the conditional moderation coefficients that compared the slopes of the association between internalization and BD at the 16th, 50th, and 84th percentiles of the distribution of BMI scores; a pattern of results replicated across the Spanish and Argentine samples. Our findings are congruent with previous research showing that BMI is robustly associated with BD (e.g., Dalley et al., 2009; Laus et al., 2011; Wojtowicz and von Ranson, 2012), and that internalization of the thin ideal may trigger BD through a process of social comparison (see Want, 2009), or cognitive discrepancy between the current and desired thinner self (Bessenoff, 2006), with the assumption being that the perceived-ideal discrepancy is likely greater among individuals with higher than lower BMIs. Although it is well known that both BMI and internalization correlate with BD, not many studies have investigated whether BMI and internalization interact to predict BD. We are aware of a small, 2 (High/Low BMI) X 2 (High/Low Internalization) study that reported a “marginal” interaction effect on BD scores (Graff et al., 2013). There is a second study that tested but did not find an interaction effect between internalization and BMI on BD (Mitchell et al., 2012). Thus, we believe the present study is the first to test and report a moderating effect of BMI on the effect of awareness of the thin ideal on BD through internalization.

An important limitation of our findings is that our study is cross-sectional and longitudinal research is more appropriate to test moderated and mediated processes. Additionally, although BD among young women is normative (Low et al., 2003), and the prevalence of BD and eating disorders is no longer thought to be circumscribed to White, Anglo-Saxon populations, we relied on two convenience, university samples from two specific cities in two different countries. That is, our findings have limited generalizability to other ages, nationalities, or even Spaniards and Argentines from other cities or regions. Conversely, our methodology also bears a few strengths that enhance the extent to which our findings contribute uniquely and substantively. Our OLS path analytic approach using bootstrapping and product of coefficients calculations to test a moderated mediation model represents notable advantages over the more traditional, causal step approach often used in psychological research (Preacher et al., 2007). Additionally, we went beyond mere comparisons of awareness, internalization, and BD averages across nationalities and examined whether a theoretically and empirically inspired mediation model replicated across nationalities, as well as whether nationality and BMI moderated the mediation model.

Whereas the cross-sectional our results precludes us from making causal inferences, our findings have important theoretical implications for informing relationships that could be investigated through longitudinal and experimental studies aimed to examines causal mechanisms leading to BD. On the one hand, we replicated in two Spanish speaking samples from two different continents the finding that awareness of the thin ideal is associated with BD through internalization of the thin ideal. We qualified this result by showing that nationality moderated the risk of BD. This pattern of results supports culture-bound theories of BD that conceptualize cultural context and cultural identity as having a fluid rather than an all or none influence in the expression of psychological phenomena (American Psychological Association, 2003). For example, the absence of significant differences between samples on SATAQ-4A and SATAQ-4I scores suggested similar awareness and vulnerability to internalize the Western ideal of female beauty in both samples. On the other hand, the interaction between internalization and nationality to predict BD reflects a stronger relationship between internalization and BD in Spanish than Argentine participants. Whereas we noted historical economic differences between Argentine and Spain, researchers could hypothesize other culturally dependent variables that may explain the interaction between nationality and internalization to predict BD. The present study focused on risk factors that may cause and mediate the emergence of BD. Perhaps future research could focus on theorizing more complex conditional mediation models of BD that could include BD as an antecedent of other eating-disorder symptomatology, and/or include hypothetical protective factors such as personality variables or individual abilities as mediators or moderators of BD and eating disorder symptoms. It is also important to note that whereas BMI appears to have influenced the degree to which internalization was associated with BD, internalization predicted BD regardless of BMI. That is, BMI emerged as an important individual factor that both correlated with BD and interacted with internalization of the thin ideal to predict BD, with the relationship being stronger among individuals with higher BMIs. Thus, future research could be aimed to understand the mechanisms or processes that govern the positive and moderating relationship between BMI and BD.

Finally, our study may have applied implications to the extent that future prevention or treatment research test interventions that aim to impact the various pathways that contribute to BD. For example, prevention interventions could aim to foster resilience against the internalization of the thin ideal, or treatments aimed to increase self-acceptance and reduce internalization of the thin ideal. Prevention and treatment interventions could also explore or target the relationship and interaction between BMI and BD to foster healthier, more realistic views and acceptance of a larger range of body shapes and sizes. For instance, Moreno-Domínguez et al. (2018), recently found that exposure to images of overweight models increased positive self-image, mood and body satisfaction, and these effects were larger in participants with healthier self-images. These authors speculated that increasing the representation of average-size and plus-size models and actors in the fashion advertisement and entertainment industries could decrease normative BD among girls and women.

## Author Contributions

SM-D, GR, and AC-B designed the study. SM-D and GR collected the data. AC-B conducted the analyses in close consultation with SM-D. SM-D and AC-B wrote the first draft of the manuscript. All authors contributed to and have approved the final manuscript.

## Conflict of Interest Statement

The authors declare that the research was conducted in the absence of any commercial or financial relationships that could be construed as a potential conflict of interest.

## References

[B1] American Psychological Association (2003). Guidelines on multi-cultural education, training, research, practice, and organizational change for psychologists. *Am. Psychol.* 58 377–402. 10.1037/0003-066x.58.5.377 12971086

[B2] Attie I. Brooks-Gunn J. (1989). Development of eating problems in adolescent girls: a longitudinal study. *Dev. Psychol.* 25 70–79. 10.1037/0012-1649.25.1.70

[B3] Baron R. M. Kenny D. A. (1986). The moderator–mediator variable distinction in social psychological research: conceptual, strategic, and statistical considerations. *J. Pers. Soc. Psychol.* 51 1173–1182. 10.1037/0022-3514.51.6.1173 3806354

[B4] Bearman S. K. Presnell K. Martinez E. Stice E. (2006). The skinny on body dissatisfaction: a longitudinal study of adolescent girls and boys. *J. Youth Adolesc.* 35 229–241. 10.1007/s10964-005-9010-9 PMC1540456 16912810

[B5] Becker A. E. Fay K. Agnew-Blais J. Guarnaccia P. M. Striegel-Moore R. H. Gilman S. E. (2010). Development of a measure of ‘acculturation’ for ethnic Fijians: methodologic and conceptual considerations for application to eating disorders research. *Transcult. Psychiatry* 47 454–488. 10.1177/1363461510382153 PMC3778982 21088103

[B6] Bessenoff G. R. (2006). Can the media affect us? Social comparison, self-discrepancy, and the thin ideal. *Psychol. Women Q.* 30 239–251. 10.1111/j.1471-6402.2006.00292.x

[B7] Bonafini B. A. Pozzilli P. (2011). Body weight and beauty: the changing face of the ideal female body weight. *Obesity Rev.* 12 62–65. 10.1111/j.1467-789X.2010.00754.x 20492540

[B8] Brewis A. A. McGarvey S. T. (2000). Body image, body size, and Samoan ecological and individual modernization. *Ecol. Food Nutr.* 39 105–120. 10.1080/03670244.2000.9991609

[B9] Bulik C. M. Wade T. D. Heath A. C. Martin N. G. Stunkard A. J. Eaves L. J. (2001). Relating body mass index to figural stimuli : population-based normative data for Caucasians. *Int. J. Obes. Relat. Metab. Disord.* 25 1517–1524. 10.1038/sj.ijo.0801742 11673775

[B10] Cafri G. Yamamiya Y. Brannick M. Thompson J. K. (2005). The influence of sociocultural factors on body image: a meta-analysis. *Clin. Psychol.* 12 421–433. 10.1093/clipsy/bpi053

[B11] Choi E. Choi I. (2016). The associations between body dissatisfaction, body figure, self-esteem, and depressed mood in adolescents in the United States and Korea: a moderated mediation analysis. *J. Adolesc.* 53 249–259. 10.1016/j.adolescence.2016.10.007 27816699

[B12] Clark L. Tiggemann M. (2008). Sociocultural and individual psychological predictors of body image in young girls: a prospective study. *Dev. Psychol.* 44 1124–1134. 10.1037/0012-1649.44.4.1124 18605839

[B13] Cooper P. J. Taylor M. J. Cooper Z. Fairburn C. G. (1987). The development and validation of the body shape questionnaire. *Int. J. Eat. Disord.* 6 485–494. 10.1002/1098-108X(198707)6:4<485::AID-EAT2260060405>3.0.CO;2-O

[B14] Corning A. F. Krumm A. J. Smitham L. A. (2006). Differential social comparison processes in women with and without eating disorder symptoms. *J. Couns. Psychol.* 53 338–349. 10.1037/0022-0167.53.3.338

[B15] Dalley S. E. Buunk A. P. Umit T. (2009). Female body dissatisfaction after exposure to overweight and thin media images: the role of body mass index and neuroticism. *Pers. Individ. Differ.* 47 47–51. 10.1016/j.paid.2009.01.044

[B16] De Gracia M. Marco M. Trujano P. (2007). Factores asociados a la conducta alimentaria en preadolescents. *Piscothema* 19 646–653. 17959121

[B17] DiNicola V. F. (1990). Anorexia multiforme: self-starvation in historical and cultural context: part ii: anorexia nervosa as a culture-reactive syndrome. *Transcult. Psychiatr. Res. Rev.* 27 245–286. 10.1177/136346159002700401

[B18] Dittmar H. Halliwell E. Stirling E. (2009). Understanding the impact of thin media models on women’s body-focused affect: the roles of thin-ideal internalization and weight related self-discrepancy activation in experimental exposure effects. *J. Soc. Clin. Psychol.* 28 43–72. 10.1521/jscp.2009.28.1.43

[B19] Dittmar H. Howard S. (2004). Ideal-body internalization and social comparison tendency as moderators of thin media models’ impact on women’s body focused anxiety. *J. Soc. Clin. Psychol.* 23 768–791. 10.1521/jscp.23.6.768.54799

[B20] Fingeret M. C. Gleaves D. H. (2004). Sociocultural, feminist, and psychological influences on women’s body satisfaction: a structural modeling analysis. *Psychol. Women Q.* 28 370–380. 10.1111/j.1471-6402.2004.00154.x

[B21] Forbes G. B. Jung J. Vaamonde J. D. Omar A. Paris L. Formiga N. S. (2012). Body dissatisfaction and disordered eating in three cultures: Argentina, Brazil, and the US. *Sex Roles* 66 677–694. 10.1007/s11199-011-0105-3

[B22] Gleaves D. H. Cepeda-Benito A. Williams T. L. Cororve M. B. Fernandez M. D. C. Vila J. (2000). Body image preferences of self and others: a comparison of Spanish and American male and female college students. *Eat. Disord.* 8 269–282. 10.1080/10640260008251236 27177300

[B23] Grabe S. Ward L. M. Hyde J. S. (2008). The role of the media in body image concerns among women: a meta-analysis of experimental and correlational studies. *Psychol. Bull.* 134 460–476. 10.1037/0033-2909.134.3.460 18444705

[B24] Graff K. A. Murnen S. K. Krause A. K. (2013). Low-cut shirts and high-heeled shoes: increased sexualization across time in magazine depictions of girls. *Sex Roles* 69 571–582. 10.1007/s11199-013-0321-0

[B25] Hayes A. F. (2013). *Introduction to Mediation, Moderation, and Conditional Process Analysis: Methodology in the Social Sciences*. New York, NY: Guilford Press.

[B26] Hayes A. F. (2018). *Introduction to Mediation, Moderation, and Conditional Process Analysis Second Edition: A Regression-Based Approach*. New York, NY: Guilford Press.

[B27] Hayes A. F. Cai L. (2007). Using heteroskedasticity-consistent standard error estimators in OLS regression: an introduction and software implementation. *Behav. Res. Methods* 39 709–722. 10.3758/bf03192961 18183883

[B28] Hayes A. F. Matthes J. (2009). Computational procedures for probing interactions in OLS and logistic regression: SPSS and SAS implementations. *Behav. Res. Methods* 41 924–936. 10.3758/BRM.41.3.924 19587209

[B29] Holmqvist K. Lunde C. Frisén A. (2007). Dieting behaviors, body shape perceptions, and body satisfaction: cross-cultural differences in argentinean and swedish 13-year-olds. *Body Image* 4 191–200. 10.1016/j.bodyim.2007.03.001 18089264

[B30] Lameiras Fernández M. Otero M. C. Castro Y. R. Prieto M. F. (2003). Hábitos alimentarios e imagen corporal en estudiantes universitarios sin trastornos alimentarios. *Int. J. Clin. Health Psychol.* 3 23–33.

[B31] Laus M. F. Costa T. M. B. Almeida S. S. (2011). Body image dissatisfaction and its relationship with physical activity and body mass index in Brazilian adolescents. *J. Bras. Psiquiatr.* 60 315–320. 10.1590/s0047-20852011000400013

[B32] Llorente E. Gleaves D. H. Warren C. S. Pérez-de-Eulate L. Rakhkovskaya L. (2015). Translation and validation of a spanish version of the sociocultural attitudes towards appearance questionnaire-4 (SATAQ-4). *Int. J. Eat. Disord.* 48 170–175. 10.1002/eat.22263 24616068

[B33] López-Guimerà G. Levine M. P. Sánchez-Carracedo D. Fauquet J. (2010). Influence of mass media on body image and eating disordered attitudes and behaviors in females: a review of effects and processes. *Media Psychol.* 13 387–416. 10.1080/15213269.2010.525737

[B34] Low K. G. Charanasomboon S. Brown C. Hiltunen G. Long K. Reinhalter K. (2003). Internalization of the thin ideal, weight and body image concerns. *Soc. Behav. Pers.* 31 81–90. 10.2224/sbp.2003.31.1.81

[B35] MacKinnon D. P. Lockwood C. M. Hoffman J. M. West S. G. Sheets V. (2002). A comparison of methods to test mediation and other intervening variable effects. *Psychol. Methods* 7 83–104. 10.1037/1082-989X.7.1.83 11928892 PMC2819363

[B36] Mann M. (2001). Globalization is (among other things) transnational, inter-national and American. *Sci. Soc.* 65 464–469. 10.1521/siso.65.4.464.17807

[B37] Marrodán D. Montero-Roblas V. Mesa S. Pacheco J. L. González M. Bejarano I. (2008). Realidad, percepción y atractivo de la imagen corporal: condicionantes biológicos y socioculturales. *Cuadernos Antropol. Etnografía* 30 15–28.

[B38] McArthur L. H. Holbert D. Peña M. (2005). An exploration of the attitudinal and perceptual dimensions of body image among male and female adolescents from six latin american cities. *Adolescence* 40 801–816. 16468673

[B39] McCabe M. P. Ricciardelli L. A. (2003). Sociocultural influences on body image and body changes among adolescent boys and girls. *J. Soc. Psychol.* 143 5–26. 10.1080/00224540309598428 12617344

[B40] Miller M. N. Pumariega A. J. (2001). Culture and eating disorders: a historical and cross-cultural review. *Psychiatry* 64 93–110. 10.1521/psyc.64.2.93.18621 11495364

[B41] Mitchell S. H. Petrie T. A. Greenleaf C. A. Martin S. B. (2012). Moderators of the internalization–body dissatisfaction relationship in middle school girls. *Body Image* 9 431–440. 10.1016/j.bodyim.2012.07.001 22858554

[B42] Moreno S. Warren C. S. Rodríguez S. Fernández M. C. Cepeda-Benito A. (2009). Food cravings discriminate between anorexia and bulimia nervosa. implications for “success” versus “failure” in dietary restriction. *Appetite* 52 588–594. 10.1016/j.appet.2009.01.011 19501754

[B43] Moreno-Domínguez S. Servián-Franco F. Reyes D. P. Cepeda-Benito A. (2018). Images of thin and plus-size models produce opposite effects on women’s body image, body dissatisfaction, and anxiety. *Sex Roles* 10.1007/s11199-018-0951-3 [E-pub ahead of print].

[B44] Olfert M. Barr M. Charlier C. Famodu O. Zhou W. Mathews A. (2018). Self-Reported vs. measured height, weight, and BMI in young adults. *Int. J. Environ. Res. Public Health* 15:2216 10.3390/ijerph15102216 PMC6210375 30314261

[B45] Petrie T. A. Greenleaf C. Martin S. (2010). Biopsychosocial and physical correlates of middle school boys’ and girls’ body satisfaction. *Sex Roles* 63 631–644. 10.1007/s11199-010-9872-5

[B46] Preacher K. J. Hayes A. F. (2004). SPSS and SAS procedures for estimating indirect effects in simple mediation models. *Behav. Res. Methods Instrum. Comput.* 36 717–731. 10.3758/BF03206553 15641418

[B47] Preacher K. J. Hayes A. F. (2008). Asymptotic and resampling strategies for assessing and comparing indirect effects in multiple mediator models. *Behav. Res. Methods* 40 879–891. 10.3758/BRM.40.3.879 18697684

[B48] Preacher K. J. Rucker D. D. Hayes A. F. (2007). Addressing moderated mediation hypotheses: theory, methods, and prescriptions. *Multivariate Behav. Res.* 42 185–227. 10.1080/00273170701341316 26821081

[B49] Preacher K. J. Selig J. P. (2012). Advantages of Monte Carlo confidence intervals for indirect effects. *Commun. Methods Meas.* 6 77–98. 10.1080/19312458.2012.679848

[B50] Quick V. Eisenberg M. E. Bucchianeri M. M. Neumark-Sztainer D. (2013). Prospective predictors of body dissatisfaction in young adults: 10-year longitudinal findings. *Emerg. Adulthood* 1 271–282. 10.1177/2167696813485738 25045599 PMC4101918

[B51] Ramos P. Rivera F. Pérez R. S. Lara L. Moreno C. (2016). Diferencias de género en la imagen corporal y su importancia en el control de peso. *Escritos Psicol.* 9 42–50. 10.5231/psy.writ.2015.1409

[B52] Ramos Valverde P. Rivera de los Santos F. J. Rodríguez M. Carmen M. (2010). Diferencias de sexo en imagen corporal, control de peso e Índice de Masa Corporal de los adolescentes españoles. *Psicothema* 22 77–83. 20100431

[B53] Rodin J. Silberstein L. Striegel-Moore R. (1984). Women and weight: a normative discontent. *Nebraska Symp. Motiv.* 32 267–307. 6398857

[B54] Schaefer L. M. Burke N. L. Thompson J. K. Dedrick R. F. Heinberg L. J. Calogero R. M. (2015). Development and validation of the sociocultural attitudes towards appearance questionnaire-4 (SATAQ-4). *Psychol. Assess.* 27 54–67. 10.1037/a0037917 25285718

[B55] Schulte S. J. Thomas J. (2013). Relationship between eating pathology, body dissatisfaction and depressive symptoms among male and female adolescents in the United Arab Emirates. *Eat. Behav.* 14 157–160. 10.1016/j.eatbeh.2013.01.015 23557812

[B56] Shin K. You S. Kim E. (2017). Sociocultural pressure, internalization, BMI, exercise, and body dissatisfaction in korean female college students. *J. Health Psychol.* 22 1712–1720. 10.1177/1359105316634450 26936503

[B57] Stice E. (1994). Review of the evidence for a sociocultural model of bulimia nervosa and an exploration of the mechanisms of action. *Clin. Psychol. Rev.* 14 633–661. 10.1016/0272-7358(94)90002-7

[B58] Stice E. (2002). Risk and maintenance factors for eating pathology: a meta-analytic review. *Psychol. Bull.* 128 825–848. 10.1037/0033-2909.128.5.825 12206196

[B59] Stice E. Mazotti L. Krebs M. Martin S. (1998). Predictors of adolescent dieting behaviors: a longitudinal study. *Psychol. Addict. Behav.* 12 195–205. 10.1037/0893-164X.12.3.195

[B60] Stice E. Orjada K. Tristan J. (2006). Trial of a psychoeducational eating disturbance intervention for college women: a replication and extension. *Int. J. Eat. Disord.* 39 233–239. 10.1002/eat.20252 16498589

[B61] Stice E. Whitenton K. (2002). Risk factors for body dissatisfaction in adolescent girls: a longitudinal investigation. *Dev. Psychol.* 38 669–678. 10.1037/0012-1649.38.5.669 12220046

[B62] Thompson J. K. Heinberg L. J. Altabe M. Tantleff-Dunn S. (1999). *Interpersonal factors: Peers, Parents, Partners, and Perfect Strangers. Exacting beauty: Theory, Assessment, and Treatment of Body Image Disturbance; Exacting Beauty: Theory, Assessment, and Treatment of Body Image Disturbance*. Washington, DC: American Psychological Association, 175–207. 10.1037/10312-006

[B63] Thompson J. K. Stice E. (2001). Thin-ideal internalization: mounting evidencce for a new risk factor for body-image disturbance and eating pathology. *Curr. Dir. Psychol. Sci.* 10 181–183. 10.1111/1467-8721.00144

[B64] Tiggemann M. Gardiner M. Slater A. (2000). “I would rather be a size 10 than have straight A’s”: a focus group study of adolescent girls’ wish to be thinner. *J. Adolesc.* 23 645–659. 10.1006/jado.2000.0350 11161330

[B65] Verplanken B. Tangelder Y. (2011). No body is perfect: the significance of habitual negative thinking about appearance for body dissatisfaction, eating disorder propensity, self-esteem and snacking. *Psychol. Health* 26 685–701. 10.1080/08870441003763246 21347977

[B66] Wade T. D. Zhu G. Martin N. G. (2011). Undue influence of weight and shape: Is it distinct from body dissatisfaction and concern about weight and shape? *Psychol. Med.* 41 819–828. 10.1017/s0033291710001066 20507670

[B67] Want S. C. (2009). Meta-analytic moderators of experimental exposure to media portrayals of women on female appearance satisfaction: social comparisons as automatic processes. *Body Image* 6 257–269. 10.1016/j.bodyim.2009.07.008 19716779

[B68] Warren C. S. Cepeda-Benito A. Gleaves D. H. Moreno S. Rodriguez S. Fernandez M. C. (2008). English and spanish versions of the body shape questionnaire: measurement equivalence across ethnicity and clinical status. *Int. J. Eat. Disord.* 41 265–272. 10.1002/eat.20492 18076088

[B69] Warren C. S. Gleaves D. H. Cepeda-Benito A. del C. F. Rodriguez-Ruiz S. (2005). Ethnicity as a protective factor against internalization of a thin ideal and body dissatisfaction. *Int. J. Eat. Disord.* 37 241–249. 10.1002/eat.20102 15822090

[B70] Williams T. L. Gleaves D. H. Cepeda-Benito A. Erath S. A. Cororve M. B. (2001). The reliability and validity of a group-administered version of the body image assessment. *Assessment* 8 37–46. 10.1177/107319110100800104 11310725

[B71] Wojtowicz A. E. von Ranson K. M. (2012). Weighing in on risk factors for body dissatisfaction: a one-year prospective study of middle-adolescent girls. *Body Image* 9 20–30. 10.1016/j.bodyim.2011.07.004 22078900

[B72] World Bank Data Help Desk (2018). *World Bank Country, and. (Lending) Groups: Historical Classification by Income in XLS Format*. Avaialble at: https://datahelpdesk.worldbank.org/knowledgebase/articles/906519

[B73] Zabinski M. F. Pung M. A. Wilfley D. E. Eppstein D. L. Winzelberg A. J. Celio A. (2001). Reducing risk factors for eating disorders: targeting at-risk women with a computerized psychoeducational program. *Int. J. Eat. Disord.* 29 401–408. 10.1002/eat.1036 11285577

